# Supplementary material for: Analyzing Barriers and Enablers for the Acceptance of Artificial Intelligence Innovations into Radiology Practice: A Scoping Review
Source: Tomography. 2023 Jul 28;9(4):1443–55. doi: 10.3390/tomography9040115 (PMC10459931; doi:10.3390/tomography9040115)
Supplement: Supplementary file 1 [file tomography-09-00115-s001.zip › tomography-2476888-supplementary.pdf]

tomography

MDPI

Systematic Review

Analyzing Barriers and Enablers for the Acceptance of Artificial Intelligence Innovations into Radiology Practice: A Scoping Review

Fatma A. Eltawil [1], Michael Atalla [1], Emily Boulos [1], Afsaneh Amirabadi [2] and Pascal N. Tyrrell [1,3,4,*]

[1] Department of Medical Imaging, University of Toronto, Toronto, ON M5S 1A1, Canada; fatma.eltawil@tyrrell4innovation.ca (F.A.E.); m2atalla@uwaterloo.ca (M.A.); emily.boulos@gmail.com (E.B.)
[2] Diagnostic Imaging Department, The Hospital for Sick Children, Toronto, ON M5G 1E8, Canada; afsaneh.amirabadi@sickkids.ca
[3] Department of Statistical Sciences, University of Toronto, Toronto, ON M5G 1Z5, Canada
[4] Institute of Medical Science, University of Toronto, Toronto, ON M5S 1A8, Canada
* Correspondence: pascal.tyrrell@utoronto.ca; Tel.: 416-978-7941

Supplementary Material

Table S1: Medline search strategy

**CAD**	
1	exp diagnosis, computer-assisted/ or exp image interpretation, computer assisted/ or exp decision making, computer-assisted/
2	computer-aided diagnosis.mp.
3	exp Radiographic Image Interpretation, Computer-Assisted/
4	exp Diagnostic Imaging/
5	exp Radiology Information Systems/
6	"Attitude of Health Personnel"/
7	exp Attitude to Computers/
8	Radiologist.mp.
9	evaluation.mp.
10	(computer-aided detection and diagnosis systems).mp. [mp=title, abstract, original title, name of substance word, subject heading word, keyword heading word, protocol supplementary concept word, rare disease supplementary concept word, unique identifier, synonyms]
11	(quality assurance and training procedures).mp. [mp=title, abstract, original title, name of substance word, subject heading word, keyword heading word, protocol supplementary concept word, rare disease supplementary concept word, unique identifier, synonyms]
12	computer-hindered.mp.
13	(junior radiologist or senior radiologist).mp. [mp=title, abstract, original title, name of substance word, subject heading word, keyword heading word, protocol supplementary concept word, rare disease supplementary concept word, unique identifier, synonyms]
14	radiologist's experience.mp.
15	laboratory to the clinic.mp.
16	1 or 2 or 3 or 4 or 5
**Artificial intelligence**	
1	exp Radiographic Image Interpretation, Computer-Assisted/ and exp Radiology/ and exp Artificial Intelligence/

2	exp Artificial Intelligence/ and exp Radiology/ and exp Decision Making/

Table S2: Embase search strategy

**CAD**	
1	exp computer assisted diagnosis/
2	computer aided diagnosis.mp.
3	exp diagnostic imaging/
4	radiology information systems.mp.
5	exp health personnel attitude
6	exp attitude to computers/
7	exp radiology/ or exp radiologist/
8	evaluation.mp. or exp evaluation study/
9	(computer-aided detection and diagnosis systems).mp. [mp=title, abstract, heading word, drug trade name, original title, device manufacturer, drug manufacturer, device trade name, keyword, floating subheading word]
10	(quality assurance and training procedures). mp. [mp=title, abstract, heading word, drug trade name, original title, device manufacturer, drug manufacturer, dovice trode name bowverd flesting cubhesding ward
11	computer-hindered.mp.
12	(Junior radiologist or senior radiologist).mp. [mp=title, abstract, heading word, drug trade name, original title, device manufacturer, drug manufacturer, device trade name, keyword, floating subheading word]
13	radiologist's experience.mp.
14	laboratory to the clinic.mp.
15	1 or 2 or 3 or 4
16	Computer-aided diagnosis in radiology.mp.
**Artificial intelligence**	
1	exp Radiographic Image Interpretation, Computer-Assisted/ and exp Radiology/ and exp Artificial Intelligence/
2	exp Artificial Intelligence/ and exp Radiology/ and exp Decision Making/
